# Phase I study of PF‐04895162, a Kv7 channel opener, reveals unexpected hepatotoxicity in healthy subjects, but not rats or monkeys: clinical evidence of disrupted bile acid homeostasis

**DOI:** 10.1002/prp2.467

**Published:** 2019-02-11

**Authors:** Michael D. Aleo, Jiri Aubrecht, Paul D. Bonin, Deborah A. Burt, Jennifer Colangelo, Lina Luo, Shelli Schomaker, Rachel Swiss, Simon Kirby, Greg C. Rigdon, Pinky Dua

**Affiliations:** ^1^ Investigative Toxicology Drug Safety Research and Development Pfizer Inc. Groton Connecticut; ^2^ Safety Biomarkers Drug Safety Research and Development Pfizer Inc. Groton Connecticut; ^3^ Medicine Design Primary Pharmacology Group Pfizer Inc. Groton Connecticut; ^4^ Compound Safety Prediction Worldwide Medicinal Chemistry Pfizer Inc. Groton Connecticut; ^5^ Global Biometrics and Data Management Pfizer Inc. Cambridge UK; ^6^ Neusentis Research Unit Pfizer Inc. Durham North Carolina; ^7^ Clinical Pharmacology Early Clinical Development Pfizer Inc. Cambridge UK; ^8^ Present address: QuatroBio, LLC Morrisville North Carolina

**Keywords:** bile acid conjugation status, bile acid homeostasis, BSEP inhibition, hepatotoxicity, PF‐04895162 (ICA‐105665)

## Abstract

During a randomized Phase 1 clinical trial the drug candidate, PF‐04895162 (ICA‐105665), caused transaminase elevations (≥grade 1) in six of eight healthy subjects treated at 300 mg twice daily for 2‐weeks (NCT01691274). This was unexpected since studies in rats (<6 months) and cynomolgus monkeys (<9 months) treated up to 100 mg/kg/day did not identify the liver as a target organ. Mechanistic studies showed PF‐04895162 had low cytotoxic potential in human hepatocytes, but inhibited liver mitochondrial function and bile salt export protein (BSEP) transport. Clinical relevance of these postulated mechanisms of liver injury was explored in three treated subjects that consented to analysis of residual pharmacokinetic plasma samples. Compared to a nonresponder, two subjects with transaminase elevations displayed higher levels of miRNA122 and total/conjugated bile acid species, whereas one demonstrated impaired postprandial clearance of systemic bile acids. Elevated taurine and glycine conjugated to unconjugated bile acid ratios were observed in two subjects, one before the onset of elevated transaminases. Based on the affinity of conjugated bile acid species for transport by BSEP, the profile of plasma conjugated/unconjugated bile acid species was consistent with inhibition of BSEP. These data collectively suggest that the human liver injury by PF‐04895162 was due to alterations in bile acid handling driven by dual BSEP/mitochondrial inhibition, two important risk factors associated with drug‐induced liver injury in humans. Alterations in systemic bile acid composition were more important than total bile acids in the manifestation of clinical liver injury and may be a very early biomarker of BSEP inhibition.

**Abbreviations**
AE: adverse event
ALT: alanine aminotransferase
AST: aspartate aminotransferase
AUC_inf_: area under the plasma concentration time profile from time zero extrapolated to infinite time
AUC_last_: area under the plasma concentration time profile from time zero to the time of last quantifiable concentration
AUC_tau_: area under the curve from the time of dosing to the next dose
BID: twice daily
BSEP: bile salt export protein
CA: cholic acid
CDCA: chenodeoxycholic acid
CIs: confidence Intervals
C‐SSRS: Columbia suicide severity rating scale
C_max_: maximum observed plasma concentration
DCA: deoxycholic acid
DILI: drug‐induced liver injury
ECG: 12 lead electrocardiograms
GCA: glycocholic acid
GCDCA: glycochenodeoxycholic acid
GGT: γ‐glutamyl transpeptidase
MedDRA: Medical Dictionary for Regulatory Activities
MRP3/4: multidrug resistance‐associated protein 3/4
MDR3: multidrug resistance protein 3
PK: pharmacokinetics
NTCP: sodium/taurocholate cotransporting polypeptide
TBIL: total bilirubin
TCA: taurocholic acid
TCDCA: taurochenodeoxycholic acid
T_max_: time of maximum concentration
t_1/2_: terminal elimination half‐life

## 1 INTRODUCTION

Severe drug‐induced liver injury (DILI) is a challenging issue for health care providers, regulators, and pharmaceutical companies. In severe cases patients may require a liver transplant or experience death. Hepatotoxic agents can be broadly classified into two categories: intrinsic hepatotoxicants (those that cause liver injury predictably in humans and animals when given in sufficiently high doses) and idiosyncratic hepatotoxicants (those that cause liver injury in “susceptible” individual humans, are more varied in their clinical presentation, and generally do not cause hepatotoxicity in animals).^1^ Hepatotoxic agents in the former category are detected in standardized nonclinical safety assessment studies and generally removed from further drug development. However, due to the low incidence of idiosyncratic hepatotoxicity, potential pharmaceutical agents in the latter category are more difficult to detect in standardized nonclinical/clinical studies and may not become evident until well after marketing approval is granted. There is a third category in practice where drug candidates cause a relatively high incidence of transaminase elevations in early clinical trials that were not detected in nonclinical safety assessment studies. Trying to mitigate these risks are the subject of many initiatives within the pharmaceutical industry^2^, ^3^, ^4^ that can vary in their approach.^5^, ^6^, ^7^, ^8^ Integrating these approaches into decisions regarding medicinal design and compound selection is important since standard animal models only predict about 55%^9^, ^10^ of human transaminase elevations.PF‐04895162 (ICA‐105665, discovered by Icagen, Inc., Durham, NC), is a novel small molecule that showed signs of efficacy for the treatment of epilepsy^11^ by opening neuronal Kv7.2/7.3 and Kv7.3/7.5 potassium channels.^12^ In nonclinical studies, only a single 7‐day exploratory toxicity study in rats showed a dose‐dependent alanine aminotransferase (ALT) elevation that was not accompanied by any histological correlate. This finding was not confirmed in a repeat 7 day study at a higher dose in rats or in longer term safety assessment studies in rats and cynomolgus monkeys of 6 and 9 months in duration, respectively. Therefore, this drug candidate advanced into clinical studies in healthy subjects and patients with epilepsy. No evidence of liver injury in healthy subjects was observed in single dose studies up to 600 mg^11^ or multiple doses up to 200 mg twice daily (BID) for 7 days.^13^ Mild/moderate transaminase elevations were noted in one of 12 subjects each at 250 mg BID and 300 mg BID PF‐04895162 for 7‐days (Pfizer data on file). However, in a 14‐day multiple dose study in healthy subjects, transaminase elevations as high as 5x the ULN were noted in six of eight subjects who received 300 mg BID PF‐04895162. This high incidence rate in a small subset of subjects led to the discontinuation of this drug candidate from further clinical development.

To investigate mechanistic factors possibly contributing to the observed liver injury, a broad suite of assays were conducted where inhibition of both mitochondrial function and BSEP transporter activity were highlighted as possible mechanisms. To establish a clinical correlation to these postulated mechanistic liabilities, that are known independent or combined drivers of liver injury, a few treated subjects from the 14‐day clinical study consented to additional biomarker examination using residual pharmacokinetic samples. Elevations in miRNA122 levels coincided with ALT elevations, confirming liver origin of ALT. Total and individual tauro‐ and glyco‐conjugated bile acids, which are purportedly preferential substrates for BSEP transport, were elevated in plasma from affected, but not unaffected subjects, suggesting some specific effect of PF‐04895162 on hepatobiliary elimination of bile acids concurrently or in advance of liver injury. Although more clinical drug examples are needed to confirm these observations as evidence of interruption of hepatobiliary transport of bile acids, resulting in liver transaminase elevation, the present investigation provided a blueprint for showing correlations between in vitro mechanistic assays with clinical findings and the need to examine total and individual bile acids species as a means to provide relevant supporting biomarker data of a mechanistically linked clinical outcome.

## 2 MATERIALS AND METHODS

### 2.1 Materials

PF‐04895162 (N‐(2‐cyclopropyl‐7‐fluoro‐4‐oxoquinazolin‐3(4*H*)‐yl)‐2‐(4‐fluorophenyl)ethanamide) (MW = 355.344) was prepared under current good manufacturing practice for nonclinical and clinical use.

### 2.2 Bioethical statements for conduct of nonclinical studies

These studies were conducted in accordance with the United States Food and Drug Administration Good Laboratory Practice Regulations, 21 Code of Federal Regulations Part 28. All animals received humane care according to the criteria outlined in the “Guide for the Care and Use of Laboratory Animals” prepared by the National Academy of Sciences and published by the National Institutes of Health (NIH publication 86‐23 revised 1985).

### 2.3 Clinical study design and clinical trial objectives

**Figure 1:**
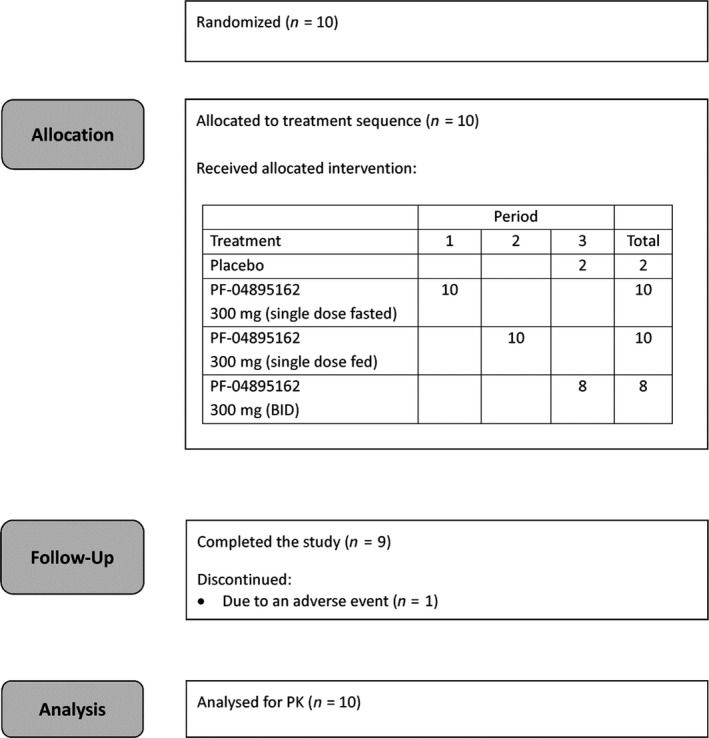
Disposition of subjects

A randomized, double‐blind, third‐party open (i.e., subject and Investigator blind, and Sponsor open), placebo‐controlled study was conducted in healthy subjects to investigate the safety, tolerability, and pharmacokinetics of single and multiple oral doses of PF‐04895162 and to characterize the effect of food on the pharmacokinetics of a single oral dose of PF‐04895162. The study was conducted in accordance with the International Conference on Harmonization guideline for Good Clinical Practice and the principles of the Declaration of Helsinki and was approved by the local independent ethics committee. All subjects gave written informed consent prior to participation in the study. The study was planned as a parallel group, dose escalation study in four cohorts of 10 subjects each, however, the study was terminated prematurely and only Cohort 1 was conducted. After an initial screening visit to confirm eligibility, subjects in Cohort 1 (Figure 1) received single oral doses of 300 mg PF‐04895162 in Period 1 (under fasting conditions) and Period 2 (under fed conditions), and then 300 mg BID PF‐04895162 or placebo for 14 days (under fasting conditions) in Period 3 (the final dose was administered on the morning of Day 14). In Period 2, subjects were given a high‐fat breakfast 25 minutes prior to dosing which had to be consumed within 20 minutes. In all periods, subjects were fasted for at least 8 hours prior to morning dosing (except for breakfast in Period 2), and received lunch and dinner approximately 4 and 9‐10 hours after the morning dose, respectively. In Period 3, subjects were randomized so that eight subjects received PF‐04895162 and two subjects received placebo. The single doses in Periods 1 and 2, and the first dose in Period 3 were each separated by 3 days. A follow‐up visit was conducted 7‐14 days after the final dose. The ClinicalTrials.gov identifier was NCT01691274.

### 2.4 Subjects

Subjects were healthy males and/or females of nonchildbearing potential, aged 18 to 55 years inclusive with body mass index of 17.5 to 30.5 kg/m^2^ and total body weight of >50 kg. Healthy was defined as no clinically relevant abnormalities identified by a detailed medical history, full physical examination, including blood pressure and pulse rate measurement, 12 lead electrocardiograms (ECGs) or clinical laboratory tests. Subjects with evidence or history of clinically significant disease (including drug allergies, but excluding untreated, asymptomatic, seasonal allergies at the time of dosing) and pregnant or nursing females were excluded from this study. Subjects with a history of seizures or conditions with a risk of seizures were also excluded. Other exclusion criteria were generally standard for healthy subject studies and included conditions affecting drug absorption, evidence of drug and/or alcohol abuse, and restrictions on the use of prescription, nonprescription, and over‐the‐counter medications and herbal remedies.

### 2.5 Clinical assessments

Safety was assessed throughout the study by monitoring adverse events, vital signs (blood pressure, pulse rate, and body temperature), 12‐lead ECGs, and physical examinations. Blood samples for safety laboratory assessments were collected prior to and 24 hours after the first single dose of PF‐04895162 (Day 1 and 2 of Period 1), and in Period 3 on Days 7, 14, and 21 (additional samples were also collected in response to emerging safety data). The Columbia suicide severity rating scale (C‐SSRS) was also conducted at screening or baseline and at the follow‐up visit. Blood samples for pharmacokinetic profiles of PF‐04895162 were collected from predose to 72 hours after each single dose (Periods 1 and 2), and up to 12 and 48 hours after morning dosing on Days 7 and 14, respectively, in Period 3. Additional trough samples were also collected on Days 5, 6, 12, and 13 of Period 3.

### 2.6 Statistical analysis used in the clinical study

The sample size of 10 subjects per cohort was chosen based on practical considerations and was considered sufficient to provide adequate safety information at each dose level. To assess the effect of food on the pharmacokinetics of PF‐04895162, natural log transformed area under the plasma concentration‐time curve (AUC) from time 0 to the last measured time point (AUClast), AUC from time 0 extrapolated to infinity (AUCinf) and maximum plasma concentration (Cmax) were analyzed using a mixed effects model with treatment (fed or fasted) as a fixed effect and subject as a random effect. Estimates of the adjusted mean differences (Fed ‐ Fasted) and corresponding 90% confidence intervals (CIs) were obtained from the model. The adjusted mean differences and 90% CIs for the differences were exponentiated to provide estimates of the ratio of adjusted geometric means (Fed/Fasted) and 90% CIs for the ratios. No formal hypothesis testing was conducted.

### 2.7 Summary methods for in vitro investigative work

Methodology for the following in vitro assays and biomarker determinations were as follows: THLE/HepG_2_ 72 hour cytotoxicity assays based on ATP depletion;^14^ mitochondrial functional assessment based on state 3/4 respiration determination in the presence of glutamate/malate^15^, ^16^ or fatty acid oxidation inhibition using palmitate;^17^ BSEP inhibition in vesicles using tritium labeled taurocholic acid (TCA);^18^ human hepatocyte imaging assay determination of cell loss, mitochondrial membrane permeability, glutathione content, and production of reactive oxygen species;^19^ miRNA‐122 determination using QuantiGene 2.0 miRNA;^20^ human multidrug resistance protein 3 inhibition/activation (MDR3, encoded by ABCB4) was determined at Biotranex based on biotransformation of *d*_9_‐choline to *d*_9_‐phosphatidylcholine in human hepatocytes;^21^ and fractionated bile acid determinations were conducted by LC‐MS/MS methodology.^22^ These various assays have been published in detail and used by us in previous investigative works.^2^, ^3^, ^21^ Examination of human sodium/taurocholate cotransporting polypeptide (NTCP, encoded by SLC10A1) was determined based on uptake of tritium labeled TCA in CHO cells and human multidrug resistance‐associated protein 3/4 (MRP3/4, encoded by ABCC3/4) transporter inhibition in vesicles obtained from HEK293 cells using tritium labeled estradiol 17 β‐D‐glucuronide and dehydroepiandrosterone sulfate as substrates, respectively. Both assays were conducted at Solvo Biotechnology (Hungary) according to their protocols.^23^, ^24^ Calculations for the Heuman index for hydrophobicity of bile acids in serum were based on the sums of bile acid hydrophobicity for each measured bile acid (AUC 0‐12 h) where larger values are considered a more hydrophobic environment than smaller values.^25^

## 3 RESULTS

### 3.1 Nonclinical safety assessment summary

A summary of liver effects along with a detailed synopsis of each nonclinical study that was used to support the Investigational New Drug Application filing along with longer term studies can be found in the supplemental information associated with this article. In brief, this compound advanced into clinical studies without any histopathological evidence of liver injury or reproducible transaminase elevations in rats and monkeys. The addition of total bile acid analysis in nonclinical safety assessment studies, although germane to this investigation retrospectively, is not routine.^26^ Absorption, distribution, metabolism, and excretion of parent and metabolites were examined in albino and pigmented male rats after administration of a single oral dose (target 15 mg/kg) of ^14^C‐labeled parent. Elimination of radioactivity occurred mostly within the first 24 hours after dosing with approximately 71 and 22% of the administered dose excreted in feces and urine, respectively. An equivalent human study was not performed.

### 3.2 Healthy subject clinical study summary

Ten subjects were enrolled and received treatment in the clinical study. All subjects were male, aged 19 to 44 years, eight subjects were Black and two subjects were White. One subject was withdrawn due to an adverse event following 300 mg BID PF‐04895162. All other subjects completed the study.

Safety: All subjects reported at least 1 adverse event during the study (Table 1). None of the adverse events were severe or serious. One subject (Subject 7) discontinued after 3 days of 300 mg BID PF‐04895162 due to clinically significant laboratory abnormalities (elevated lipase and amylase) which were investigated in response to the subject reporting mild abdominal pain. Laboratory tests on Day 3 of Period 3 showed a lipase value of 434 (IU/L) (ULN = 60 IU/L) and an amylase value of 179 (IU/L) (ULN = 136 IU/L). These values were considered treatment‐related by the Investigator and the subject was withdrawn from study. The only adverse events reported by more than one subject in any treatment group were increased transaminases (six subjects), increased amylase, increased flatulence, and decreased appetite (two subjects each), all of which occurred following 300 mg BID PF‐04895162.

**Table 1:** Summary of adverse events‐all causalities (treatment‐related)

	Placebo	PF‐04895162 Dose		
		300 mg (Single Dose, Fasted)	300 mg (Single Dose, Fed)	300 mg BID
	N = 2	N = 10	N = 10	N = 8
Number of Subjects with AEs	2 (0)	1 (1)	2 (0)	8 (6)
Number of Subjects with severe AEs	0	0	0	0
Number of Subjects with serious AEs	0	0	0	0
Number of Subjects discontinued due to AEs	0	0	0	1 (1)
Number of Subjects with the most common^a^ AEs:				
Transaminases increased	0	0	0	6 (6)
Amylase increased	0	0	0	2 (2)
Flatulence	0	0	0	2 (1)
Decreased appetite	0	0	0	2 (0)

AEs, adverse events; MedDRA, Medical Dictionary for Regulatory Activities; N, Total number of evaluable subjects.

a Most common is defined as any AE reported by at least two subjects in any treatment group. Subjects were counted only once per treatment in each row. MedDRA (v15.1) coding dictionary applied.

**Figure 2:**
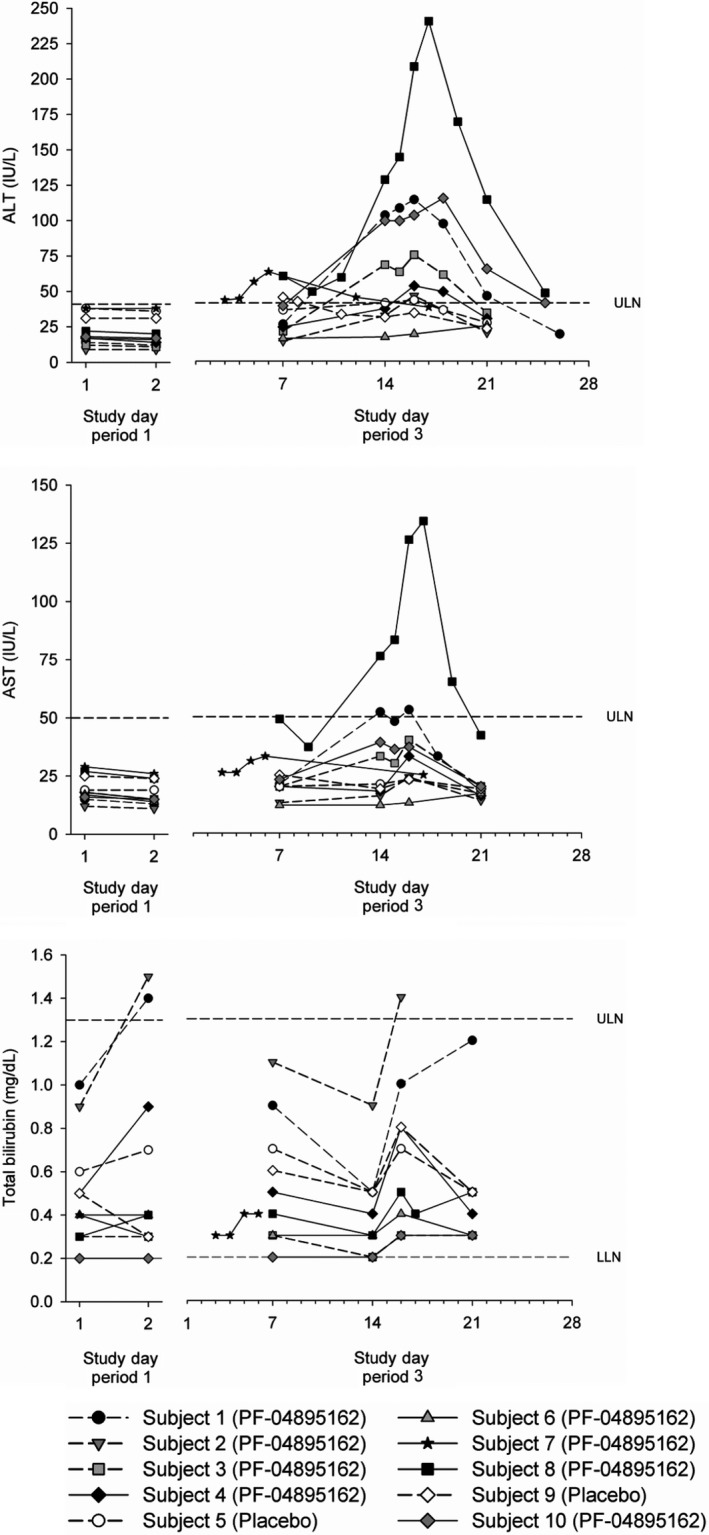
Individual ALT, AST, and Total Bilirubin concentrations after single dose (Period 1) and multiple dose PF‐04895162 at 300 mg BID (Period 3) after an overnight fast. Note: All subjects received PF‐04895162 in Period 1, whereas Subjects 5 and 9 received placebo in Period 3 only

On Day 14, six of the remaining seven subjects who finished Period 3 and received 300 mg BID PF‐04895162 had elevations in ALT of which three were classed as Grade 1 (>1.25 to 2.5x ULN), two were classed as Grade 2 (>2.5 to 5x ULN), and one was classed as Grade 3 (>5 to 10x ULN)(Table 2, Figure 2). The subject with the Grade 3 ALT elevation (5.6x ULN; Subject 8) also had a Grade 2 increase in AST (2.7x ULN), a mild elevation of GGT (1.4x ULN starting on Day 14), elevations in amylase (starting on Day 16) with no elevation of ALK PHOS. Two subjects (Subjects 1 and 2) had a Grade 1 (>1 to 1.5x ULN) increase in total bilirubin. The one subject (Subject 7) that withdrew from the study before the first scheduled assessment also had Grade 1 (>1.25 to 2.5x ULN) elevations in ALT. These elevations in transaminases/bilirubin were asymptomatic and resolved on cessation of dosing without further intervention. There were no other clinically relevant findings related to laboratory safety tests (including hepatitis panel and HIV), or other safety assessments including C‐SSRS.

**Table 2:** Summary of liver function tests abnormalities

Lab tests	Treatment	Grade			
		1	2	3	4
ALT	PF‐04895162	3	2	1	0
AST	PF‐04895162	0	1	0	0
TBIL	PF‐04895162	2	0	0	0

For ALT, AST: Grade 1, >1.25 ‐ 2.5 ×  ULN; Grade 2, >2.5 ‐ 5 ×  ULN; Grade 3, >5 ‐ 10 ×  ULN; Grade 4, >10 × ULN. For total Bilirubin: Grade 1, >1 ‐ 1.5 ×  ULN; Grade 2, >1.5 ‐ 2.5 ×  ULN; Grade 3, >2.5 ‐ 5 ×  ULN; Grade 4, >5 × ULN.

ALT, Alanine Aminotransferase; AST, Aspartate Aminotransferase; TBIL, Total Bilirubin.

### 3.3 Pharmacokinetics of PF‐04895162

Peak concentrations of PF04895162 were achieved on Days 7 and 14 at a median of 2 hours postdose, which was earlier than the median observed following a single dose (5 hours under fasting conditions) (Table 3). PF‐04895162 exposure increased from single to multiple dosing with accumulation ratios for C_max_ and AUC_tau_ on Day 14 of 1.7 and 1.6, respectively. The highest mean C_max_ (observed on Day 7) was 9432 ng/mL which is equivalent to 26.5 μmol/L (total concentration). The predicted accumulation ratio to estimate linearity (i.e., ratio of AUC_tau_ on Day 14 to AUC_inf_ on Day 1) was less than 1 (0.826) suggesting that PF‐04895162 may have nonlinear (time‐dependent) pharmacokinetics. Mean terminal half‐life was similar after single and multiple dosing (approximately 8 hours). A high‐fat meal appeared to affect the rate but not the extent of PF‐04895162 absorption as C_max_ doubled in the presence of food, whereas the 90% confidence intervals for the geometric mean ratio for AUC_inf_ were within the accepted bounds for bioequivalence (i.e., 80 to 125%) (Table 4).

**Table 3:** Summary of plasma PF‐04895162 pharmacokinetic parameter values following single and multiple oral doses

Parameter^a^, Units	Period 1 300 mg Fasted	Period 2 300 mg Fed	Period 3 300 mg BID Fasted	
	Day 1 (N = 10)	Day 1 (N = 10)	Day 7 (N = 7)	Day 14 (N = 7)
C_max_, ng/mL	4510 (30)	9152 (29)	9432 (21)	8378 (26)
C_max_, μmol/L	12.7	25.8	26.5	23.6
T_max_, h	5.00 (1.00‐6.02)	4.00 (2.00‐8.02)	2.00 (1.00‐6.00)	2.00 (0.500‐6.03)
AUC_tau_, ng.h/mL	40770 (28)	NR	85250 (20)	71160 (24)
t_&frac12;_, h	7.881 ± 3.20	5.208 ± 1.17	NA	8.043 ± 3.29
R_ac_	NA	NA	NA	1.594 (22)
R_ac_,C_max_	NA	NA	NA	1.660 (29)
Rss	NA	NA	NA	0.826 (21)

AUC_tau_, area under the plasma concentration‐time profile from over the dosing interval (12 h); C_max_, maximum plasma concentration; CV, Coefficient of variation; N, Number of subjects; NA, Not Applicable (t_&frac12;_, R_ac_, R_ac_,C_max_, and R_ss_ were determined only for Day 14); SD, Standard deviation; T_max_, time to maximum plasma concentration in hours (h); t_&frac12;_, terminal half‐live, R_ac_, accumulation ratio (AUC_tau,day14_/AUC_tau,day1_); R_ac_, C_max_, accumulation ratio (C_max,day14_/C_max,day 1_); R_ss_, accumulation at steady state (AUC_tau,day14_/AUC_inf,day 1_).

a Values are presented as geometric mean along with their %Geometric CV in parenthesis (%Geometric CV) for all except: median (range) for T_max_, and arithmetic mean ± SD for t_&frac12;_.

**Table 4:** Statistical summary of treatment comparisons (Fed vs Fasted) for plasma PF‐04895162

Parameter (Units)	Ratio (90% Confidence Interval) of Adjusted Means (%)
AUC_inf_ (ng.h/mL)	113.27 (102.99, 124.58)
AUC_last_ (ng.h/mL)	116.85 (106.90, 127.73)
C_max_ (ng/mL)	202.92 (178.28, 230.95)

AUC_inf,_ area under the plasma concentration‐time profile from time 0 extrapolated to infinite time; AUC_last_, area under the plasma concentration‐time profile from time 0 to the time of the last quantifiable concentration; C_max_, maximum plasma concentration; vs, versus. Numbers in parenthesis are the 90% confidence intervals of adjusted means.

### 3.4 Evaluation of potential hepatotoxicity mechanisms

Since hepatotoxicity was not identified as a hazard in nonclinical studies, we performed a series of in vitro mechanistic studies. PF‐04895162 did not display potent cytotoxic properties in THLE and HepG_2_ cell lines (IC_50_ ~192 and 130 μmol/L after 72 hours, respectively) or in human hepatocytes (AC_50_ for cell loss at 48 hours was >125 μmol/L based on results in three assessments in two different human hepatocyte lots (LBN and HU4165). Justification and utilization of human Cmax exposure based on total values versus free were reported by us previously.^3^ Total C_max_ exposures in human subjects (26.5 μmol/L from Table 3) were at least fivefold below the cytotoxic thresholds identified in liver cell lines and human hepatocytes. BSEP inhibition in Hi5 vesicles was noted with an IC_50_ = 106 μmol/L (N = 4, geometric mean), whereas also displaying multiple mitochondrial effects (IC_50_ ~10 μmol/L for inhibition of glutamate/malate respiration, ~70 μmol/L for impaired palmitate fatty acid oxidation, and >100 μmol/L for uncoupling activity, N = 2). Mitochondrial respiratory reserve was also compromised in human hepatocytes treated with PF‐04895162 at concentrations >11 μmol/L for 25 minutes (data not shown). Combined inhibition of mitochondria and human BSEP transport prompted us to examine clinical samples from this clinical trial more closely based on previous findings by us regarding the association of clinical liver injury with drugs that are dual BSEP and mitochondrial inhibitors.^2^ Deeper examination of possible interactions with other hepatic bile acid transporters showed weak inhibition of MRP4 (IC_50_ = 121 μmol/L) that was similar to BSEP inhibition, no inhibitory effect on MRP3, partial inhibition of NTCP (30% at 250 μmol/L), and stimulation of MDR3 activity in human hepatocytes (200 μmol/L, N = 3).

### 3.5 Examination of residual plasma pharmacokinetic samples for biomarkers of liver injury

Three of eight treated healthy subjects consented for additional analyses of residual plasma PK samples for biomarkers of liver injury (e.g., miRNA122, total, and fractionated bile acids). Subject 8 had the highest ALT elevation (maximum value during the course of study of 240 IU/L or 5.85x ULN) by Day 7, Subject 10 had a mid‐level ALT response (maximum value during the course of study of 115 IU/L or 2.8x ULN) by Day 14, whereas Subject 6 did not demonstrate any ALT elevations by Day 14, the last day of dosing (Figure 2).

**Figure 3:**
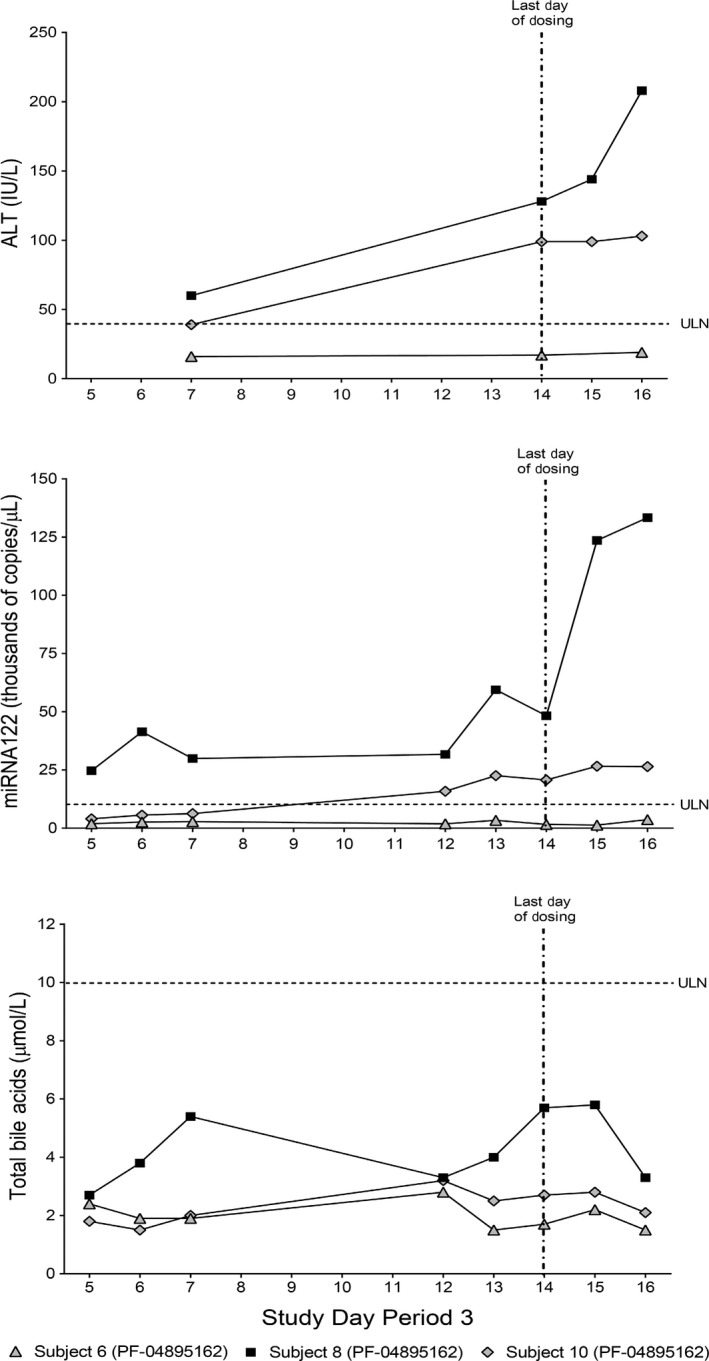
Time‐course analysis of individual patient samples from multiple dose PF‐04895162 at 300 mg BID (Period 3) for serum alanine aminotransferase (ALT) (upper panel, data from Figure 1) and who reconsented pharmacokinetic plasma samples for miRNA122 (middle panel) and total bile acids (lower panel) analysis. Samples were obtained before dose administration under fasted conditions

Serum ALT levels from the three subjects that consented to additional investigations are redisplayed from Figure 2 for illustrative comparisons (Figure 3, upper panel). Using PK samples consented for exploratory biomarker examination, we showed that plasma miRNA122 was elevated (>ULN) by Day 5 in Subject 8 and by Day 12 in Subject 10 (Figure 3, middle panel), confirming liver specificity of the elevated ALT response (Figure 3, upper panel). Both ALT and mRNA122 remained elevated 2 days after discontinuation of drug on Day 14 before returning to normal values. Total bile acids were likewise visibly higher for Subject 8 compared to other treated subjects (Figure 3, lower panel) and returned to values similar to other treated subjects within 2 days after discontinuation of drug (Day 14, morning dose administered). Although total bile acid levels were stratified between treated subjects, these levels were within normal range.

**Figure 4:**
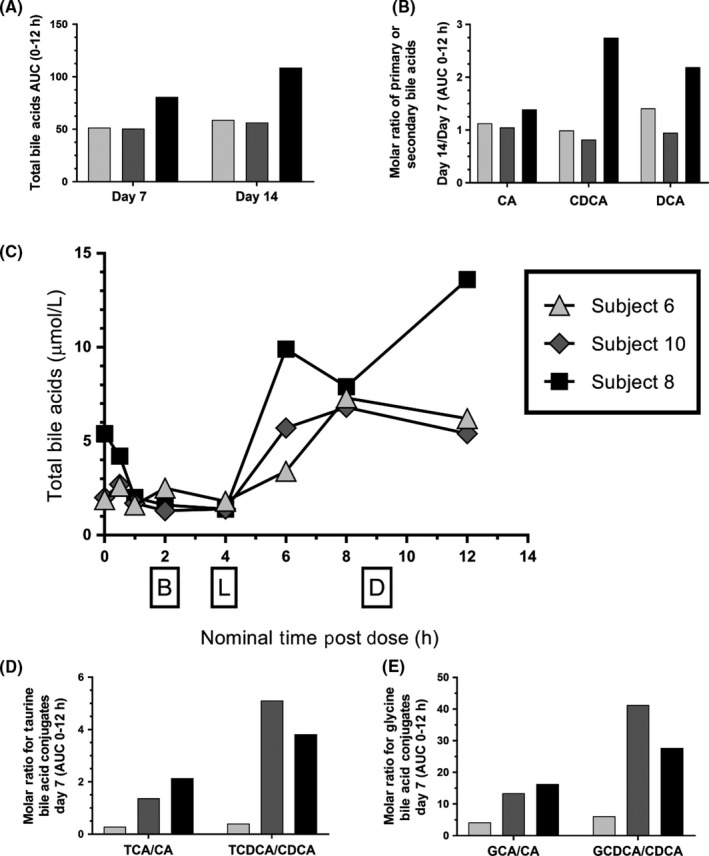
Time course of bile acid species from individual patient samples acquired from multiple dose PF‐04895162 at 300 mg BID (Period 3). Plasma samples used for pharmacokinetic analysis were reconsented for total and fractionated bile acid analysis. (A) Differences in total bile acids between treated subjects on Day 7 and 14 during a 12 h AUC time course profile (AUC 0‐12 h). (B) Ratio of unconjugated primary/secondary bile acids (CA, cholic acid; CDCA, chenodeoxycholic acid; and DCA, deoxycholic acid) Day 14/Day 7 (AUC 0‐12 h) between treated subjects. (C) Time course of total bile acids as a function of dose (open arrow) and meal (closed arrow, B, breakfast; L, lunch; D, dinner) administration on Day 7 between treated subjects. Following an 8 h fast, study medication was administered approximately one tablet every 12 h starting at approximately 0900 h on Day 1 and concluding with a morning administration on Day 14. Subjects were dosed in the morning and evening for 14 days (morning only on Day 14). Food was withheld from subjects for 2 h post morning and evening doses in a BID schedule. Subjects were fasted overnight and for 2 h prior to evening dosing. (D) Ratio of taurine conjugated to unconjugated bile acid species (TCA, taurocholic acid; CA, cholic acid; TCDCA, taurochenodeoxycholic acid; CDCA, chenodeoxycholic acid) between treated subjects. (E) Ratio of glycine conjugated to unconjugated bile acid species (GCA, glycocholic acid; CA, cholic acid; GCDCA, glycochenodeoxycholic acid; CDCA, chenodeoxycholic acid) between treated subjects. Except for Panel C total bile acids and respective ratios are based on a 12 h AUC after the morning dose with the following unit value in μmoles.h/L. Shading in bar graphs represent specific patients (light gray = Subject 6, medium gray = Subject 10, black = Subject 8)

Differential responses between treated subjects were also observed in the plasma profile of bile acids. After the first daily dose, total bile acids in systemic circulation on Day 7 (AUC 0‐12 hours) and Day 14 (AUC 0‐12 hours) were elevated in Subject 8 compared to Subjects 10 and 6 (Figure 4A). By Day 14, Subject 8 also displayed elevated levels of the more hydrophobic unconjugated bile acid species (chenodeoxycholic acid‐CDCA and deoxycholic acid‐DCA compared to cholic acid‐CA) in systemic circulation relative to Day 7 and other treated subjects (Figure 4B). On study days where we could examine time‐course effects, it was noticed that Subject 8 did not appear to clear total bile acids from systemic circulation after the midday and last meal on Day 7 (Figure 4C) as effectively as the other two subjects with a possible effect on Subject 10 after the midday meal. At this time there was also a noteworthy change in conjugated bile acids species across treated subjects. Subject 8 showed elevated bile acid molar ratios of TCDCA/CDCA relative to TCA/CA (Figure 4D) and GCDCA/CDCA relative to GCA/CA on Day 7 (Figure 4E) when ALT was 1.5x the ULN. Subject 10 showed a similar effect on bile acid molar ratios, in the absence of ALT elevations on Day 7, whereas Subject 6 was unaffected. On Day 7 the hydrophobicity index of serum bile acids were similar across subjects (0.47, 0.45, and 0.40 (AUC 0‐12 h) for Subject 6, 10, and 8, respectively). Therefore, by Day 7 there was a specific effect on the molar ratios of conjugated to unconjugated bile acid species in the absence of changes in the serum bile acid hydrophobicity index that occurred in Subject 10 before transaminase elevations were evident on Day 14. This is noteworthy since GCDCA and TCDCA are highly preferred glycine‐ and taurine‐conjugated bile acids substrates for BSEP transport compared to TCA and GCA based on intrinsic clearance values in isolated vesicles,^27^ suggestive of a specific effect on BSEP efflux function. Differences in drug exposure could not explain individual susceptibility as the subject with the highest ALT values (Subject 8) had PF‐04895162 exposures that were within the range of the rest of the treated group (C_max_ and AUC_tau_ on Day 7 for Subject 8 were 10 800 ng/mL and 93 000 ng.h/mL, respectively, compared to ranges of 7070 to 13 200 ng/mL for C_max_ and 69 600 to 121 000 ng.h/mL for AUC_tau_ in the treated cohort).

## 4 DISCUSSION

Drug candidates, that show no signs of liver injury potential in nonclinical studies of short and long duration, can progress to human trials where sometimes they demonstrate transaminase elevations within a short time frame following multidose administration in Phase 1. This occurred with PF‐04895162, a drug candidate for treating epilepsy. In nonclinical regulatory studies there were no reproducible signs of liver injury either through examination of standard biomarkers (ALT/AST) or histopathology in rats and cynomolgus monkeys treated up to 6 and 9 months, respectively. Systemic exposure in monkeys at multiple dose levels, as measured by total Cmax, were equivalent to or exceeded that achieved in this clinical study where transaminase elevations were noted in all but one treated healthy subject (Subject 6).

Examination of PF‐04895162 using mechanistic assays of liver injury potential, as previously illustrated,^3^ highlighted two possible mechanistic risk factors, BSEP transport, and mitochondrial inhibition. This combination is highly associated with liver injury in marketed and withdrawn drugs.^2^ Since residual safety lab samples were not available from this study we resorted to investigating retained clinical PK samples from a subset of subjects enrolled in the study after obtaining additional consent. Perturbations of total and/or fractionated bile acids were observed at an individual level in treated subjects who showed signs of liver injury as measured by ALT/AST and miRNA122 elevations. For example, Subject 8 had the highest systemic total bile acid levels (AUC 0‐12 h, Day 7 and Day 14), unconjugated hydrophobic bile acids CDCA and DCA (AUC 0‐12 h, ratio of Day 14/Day 7), elevated ratios of tauro‐ and glyco‐conjugated bile acids compared to unconjugated bile acids (Day 7), and did not efficiently clear systemic bile acids after meals (Day 7). This subject developed the highest levels of ALT/AST and miRNA122 of the other treated subjects (See Figure 3). Subject 10 showed disturbances in tauro‐ and glyco‐conjugated bile acid ratios that occurred before a milder ALT and miRNA122 elevation compared to Subject 8. Subject 6 showed no relative changes across time in total or fractionated bile acids levels and did not exhibit elevations in ALT/AST or miRNA122.

Given the current clinical study design and the fact that further exploratory liver biomarker work was only conducted on consented residual PK samples, it is not confidently known whether perturbations in systemic total and conjugated bile acids, especially noted after meals in Subject 8, is truly associated, let alone, causal of liver injury in this cohort of healthy subjects. This initial observation is compelling in that conjugated bile acids with the highest uptake and intrinsic clearance for human BSEP vesicle transport (TCDCA and GCDCA)^27^ are most affected for the highly (Subject 8) and modestly affected (Subject 10) subject (Figure 4). Their presence in the systemic circulation, especially in the absence of transaminase and miRNA122 elevations with Subject 10 on Day 7, suggests impaired hepatobiliary bile acid transport by BSEP^28^ leading to increased residence time and therefore conjugation of primary bile acids within the liver^29^ followed by sinusoidal efflux through MRP3/4. Enhanced intestinal reabsorption of conjugated bile acids by passive and active mechanisms^30^ cannot be ruled out at this time. Enrichment of GCDCA and TCDCA in the systemic circulation occurs under multiple cholestatic disease states in humans^31^, ^32^, ^33^, ^34^ and these specific conjugated bile acids appear to play a role in the cytotoxic and mitochondrial destructive effects of bile acids to hepatocytes,^34^, ^35^, ^36^ especially in combination with each other at millimolar in vitro exposures.^37^ This bile acid speciation phenomenon has been observed in rats treated with troglitazone 38 and TAK‐875 39 and can be observed before significant total bile acid and transaminase elevations. The former effect, if true, suggests that even moderate inhibitors of BSEP (IC_50_ = 106 μmol/L in human vesicles) with more potent mitochondrial liabilities may pose clinical risk for transaminase elevations when the ratio of systemic Cmax to BSEP vesicle IC_50_ are 0.12x initially and approach 0.22‐0.25x at steady state (see Table 3). Morgan et al.^40^ has shown with a larger collection of marketed drugs that a ratio of a drug's total systemic steady‐state exposure to BSEP IC_50_ value >0.1x highlights an enhanced risk area associated with human DILI.^41^

This compound was further modeled using DILIsym^®^ software due to complexities of trying to understand the contribution of BSEP and/or mitochondrial inhibition with drug exposure relationships with the manifestation of clinical DILI. The results of the analysis are the subject of another manuscript where it was confirmed by modeling that the combined effects of mitochondrial and BSEP inhibition could drive transaminase elevations under these clinical exposure conditions (Shoda et al., in preparation). DILIsym^®^ modeling has been conducted with other, more potent BSEP inhibitors recently like AMG 009^42^ and TAK‐875.^43^ However, in neither case has elevated total and/or fractionated bile acids been reported in these individual clinical cases of hepatotoxicity due, most likely to lack of clinical samples to examine these effects, even though this phenomenon has been demonstrated in nonclinical species treated with the same drug.^44^, ^45^

Potent BSEP inhibition by various drugs has been associated as a mechanism of liver injury in humans.^40^ In cases like bosentan, elevations in total bile acids preceded transaminase elevations at high administered doses.^46^ Subsequent analysis showed bosentan was a potent BSEP inhibitor (~12 μmol/L) and could raise total bile acids in rats.^46^ While the association between high total serum bile acids preceding transaminase elevations is strong, it is interesting to note that genetic analysis in another clinical study showed a polymorphism in CYP2C9*2 (*2/rs1799853), and not BSEP (ABCB11, rs2287622), was highly associated with bosentan‐induced transaminase elevations^47^ despite known associations of BSEP polymorphisms with liver injury.^48^ Regardless, potent BSEP inhibition, along with inhibition of other hepatic transporters, has been implicated in two recent clinical DILI examples. For example, BSEP inhibition was later implicated as causing liver injury in humans by AMG 009, an adverse finding that was not detected in preclinical safety studies.^40^ A clinical dose of 100 mg BID led to asymptomatic transaminase elevations, but is a 10‐fold more potent inhibitor of human BSEP (11.5 μmol/L) than PF‐04895162 (106 μmol/L). AMG 009 also affects other hepatic transporters involved in the regulation of hepatic bile acids (e.g., MRP2 (stimulation, followed by inhibition, no IC_50_ generated), MRP3 (1.1 μmol/L), and MRP4 (13.5 μmol/L)). Recent work with TAK‐875, an agent that caused clinical hepatotoxicity in a small number of patients at 50 mg once daily during a Phase 3 clinical study,^49^ also showed similar potencies across numerous hepatic transporters involved in bile acid transport.^50^ Unfortunately, none of these studies had clinical samples which could be used to interrogate the possible causal effect relationship between BSEP inhibition as a purported mechanism driving liver injury with evidence in affected subjects or patients. The uniqueness of this work is based on examining the availability of some clinical samples to corroborate the mechanistic work conducted in vitro. A recent clinical review article on behalf of the International Transporter Consortium suggests that an exposure margin approach, not intrinsic potency of BSEP, is needed to understand the potential hepatotoxicity liability of a drug in development,^41^ a recommendation borne out by examination of this small clinical study only made possible by access to clinical samples. This incident prompted the addition of standardized language in early clinical protocols to collect, retain and examine serum samples, leftover from the conduct of safety labs, for exploratory safety biomarker work in with subject consent in advance of the clinical trial start. Only in this way can we truly establish whether drug‐induced liver injury is causally or only associated with alterations in bile acid homeostasis and the potential use of bile acids as surrogate markers.^51^

In summary, the incidence of transaminase elevations in human healthy subjects treated with PF‐04895162 led to its termination from clinical development before its therapeutic potential could be fully examined. Although preclinical safety assessment studies did not highlight the liver as a target organ, retrospective investigation using in vitro assays designed to detect potential mechanisms of liver injury demonstrated combined BSEP and mitochondrial inhibition as potential mechanisms that are recognized as dual risk factors for human DILI.^2^ In our historical experience, for drug candidates that entered into clinical development without evidence of adverse liver injury in preclinical safety studies, 74% had BSEP inhibition properties where 55% also had mitochondrial inhibition/uncoupling activity (unpublished data). It remains to be determined why these risk factors may become apparent in early clinical trials vs postmarketing events. Weak inhibition of BSEP and MRP4 also correlated with a genetic analysis that showed polymorphisms in BSEP and MRP4 were also associated with both absolute and increased ALT levels in these responders.^52^ Examination of residual pharmacokinetic samples showed a temporal association between elevations in ALT with early retention of total and/or tauro/glyco‐conjugated bile acids, possibly due to combined mitochondrial and BSEP inhibition as the conjugated bile acids found in systemic circulation are high affinity bile acid substrates for BSEP. Although fasting total bile acids remained within the normal range (<10 μmol/L), a cytotoxic bile acid milieu probably existed within the liver based on GCDCA and TCDCA levels the systemic bile acid profile. This suggested a potential causal relationship for the transaminase elevations observed in responders which was further explored using DILIsym^®^ (Shoda et al., in preparation). Although PF‐04895162 was not a “potent” BSEP inhibitor, bile acid speciation (conjugation status) in terms of systemic retention of GCDCA and TCDCA over other precursor bile salt species reflected a “cholestatic” profile 32, 33, 34 also consistent with drug‐induced BSEP inhibition.

## DISCLOSURE

The authors report that this work did not receive external public or private foundation funding for this project. The study was sponsored by Pfizer. Authors are either former or current employees of Pfizer and may continue to hold stock or other equity positions with the company.

## References

[1] Chalasani NP , Hayashi PH , Bonkovsky HL , Navarro VJ , Lee WM , Fontana RJ and Practice Parameters Committee of the American College of G . ACG Clinical Guideline: the diagnosis and management of idiosyncratic drug‐induced liver injury. Am J Gastroenterol 2014;950‐966; quiz 967. 24935270 10.1038/ajg.2014.131

[2] Aleo MD , Luo Y , Swiss R , Bonin PD , Potter DM , Will Y . Human drug‐induced liver injury severity is highly associated with dual inhibition of liver mitochondrial function and bile salt export pump. Hepatology. 2014;1015‐1022. 24799086 10.1002/hep.27206

[3] Shah F , Leung L , Barton HA , et al. Setting clinical exposure levels of concern for drug‐induced liver injury (DILI) using mechanistic in vitro assays. Toxicol Sci. 2015;500‐514. 26206150 10.1093/toxsci/kfv152

[4] Thompson RA , Isin EM , Li Y , et al. In vitro approach to assess the potential for risk of idiosyncratic adverse reactions caused by candidate drugs. Chem Res Toxicol. 2012;1616‐1632. 22646477 10.1021/tx300091x

[5] Dykens JA , Will Y . The significance of mitochondrial toxicity testing in drug development. Drug Discov Today. 2007;777‐785. 17826691 10.1016/j.drudis.2007.07.013

[6] Greene N , Fisk L , Naven RT , Note RR , Patel ML , Pelletier DJ . Developing structure‐activity relationships for the prediction of hepatotoxicity. Chem Res Toxicol. 2010;1215‐1222. 20553011 10.1021/tx1000865

[7] Khetani SR , Kanchagar C , Ukairo O , et al. Use of micropatterned cocultures to detect compounds that cause drug‐induced liver injury in humans. Toxicol Sci. 2013;107‐117. 10.1093/toxsci/kfs326 23152190

[8] Wager TT , Kormos BL , Brady JT , et al. Improving the odds of success in drug discovery: choosing the best compounds for in vivo toxicology studies. J Med Chem. 2013;9771‐9779. 24219752 10.1021/jm401485p

[9] Greaves P , Williams A , Eve M . First dose of potential new medicines to humans: how animals help. Nat Rev Drug Discov. 2004;226‐236. 15031736 10.1038/nrd1329

[10] Olson H , Betton G , Robinson D , et al. Concordance of the toxicity of pharmaceuticals in humans and in animals. Regul Toxicol Pharmacol. 2000;56‐67. 11029269 10.1006/rtph.2000.1399

[11] Kasteleijn‐Nolst Trenite DG , Biton V , French JA , et al. Kv7 potassium channel activation with ICA‐105665 reduces photoparoxysmal EEG responses in patients with epilepsy. Epilepsia. 2013;1437‐1443. 23692516 10.1111/epi.12224 PMC3838622

[12] Roeloffs R , Wickenden AD , Crean C , et al. In vivo profile of ICA‐27243 [N‐(6‐chloro‐pyridin‐3‐yl)‐3,4‐difluoro‐benzamide], a potent and selective KCNQ2/Q3 (Kv7.2/Kv7.3) activator in rodent anticonvulsant models. J Pharmacol Exp Ther. 2008;818‐828. 10.1124/jpet.108.137794 18577704

[13] Bialer M , Johannessen SI , Levy RH , Perucca E , Tomson T , White HS . Progress report on new antiepileptic drugs: a summary of the Eleventh EILAT Conference (EILAT XI). Epilepsy Res. 2013;2‐30. 23219031 10.1016/j.eplepsyres.2012.10.001

[14] Shah F , Louise‐May S , Greene N . Chemotypes sensitivity and predictivity of in vivo outcomes for cytotoxic assays in THLE and HepG2 cell lines. Bioorg Med Chem Lett. 2014;2753‐2757. 24794102 10.1016/j.bmcl.2014.04.039

[15] Hynes J , Marroquin LD , Ogurtsov VI , et al. Investigation of drug‐induced mitochondrial toxicity using fluorescence‐based oxygen‐sensitive probes. Toxicol Sci. 2006;186‐200. 16638925 10.1093/toxsci/kfj208

[16] Hynes J , Nadanaciva S , Swiss R , Carey C , Kirwan S , Will Y . A high‐throughput dual parameter assay for assessing drug‐induced mitochondrial dysfunction provides additional predictivity over two established mitochondrial toxicity assays. Toxicol In Vitro. 2013;560‐569. 23147640 10.1016/j.tiv.2012.11.002

[17] Rogers GW , Nadanaciva S , Swiss R , Divakaruni AS , Will Y . Assessment of fatty acid beta oxidation in cells and isolated mitochondria. Curr Protoc Toxicol 2014;25.3.1‐25.319. 10.1002/0471140856.tx2503s60 24865647

[18] Marroquin LD , Bonin PD , Keefer J , Schroeter T . Assessment of bile salt export pump (BSEP) inhibition in membrane vesicles using radioactive and LC/MS‐based detection methods. Curr Protoc Toxicol 2017;14.14.1‐14.1420. 10.1002/cptx.15 28146280

[19] Lapham K , Novak J , Marroquin LD , et al. Inhibition of hepatobiliary transport activity by the antibacterial agent fusidic acid: Insights into factors contributing to conjugated hyperbilirubinemia/cholestasis. Chem Res Toxicol. 2016;1778‐1788. 27676153 10.1021/acs.chemrestox.6b00262

[20] Affymetrix (2011) Preparation of plasma or exosome lysates for use in QuantiGene 2.0 miRNA assays, in Technical Note, ThermoFisher.

[21] Aleo MD , Shah F , He K , Bonin PD , Rodrigues AD . Evaluating the role of multidrug resistance protein 3 (MDR3) inhibition in predicting drug‐induced liver injury using 125 pharmaceuticals. Chem Res Toxicol. 2017;1219‐1229. 28437613 10.1021/acs.chemrestox.7b00048

[22] Luo L , Aubrecht J , Li D , et al. Assessment of serum bile acid profiles as biomarkers of liver injury and liver disease in humans. PLoS ONE. 2018;e0193824. 29513725 10.1371/journal.pone.0193824 PMC5841799

[23] Heredi‐Szabo K , Kis E , Krajcsi P . The vesicular transport assay: validated in vitro methods to study drug‐mediated inhibition of canalicular efflux transporters ABCB11/BSEP and ABCC2/MRP2. Curr Protoc Toxicol 2012;23.4.1‐16. 10.1002/0471140856.tx2304s54 23169269

[24] Jani M , Beery E , Heslop T , et al. Kinetic characterization of bile salt transport by human NTCP (SLC10A1). Toxicol In Vitro. 2018;189‐193. 10.1016/j.tiv.2017.10.012 29024779

[25] Heuman DM . Quantitative estimation of the hydrophilic‐hydrophobic balance of mixed bile salt solutions. J Lipid Res. 1989;719‐730. 2760545

[26] Boone L , Meyer D , Cusick P , et al. Selection and interpretation of clinical pathology indicators of hepatic injury in preclinical studies. Vet Clin Pathol. 2005;182‐188. 16134065 10.1111/j.1939-165x.2005.tb00041.x

[27] Hayashi H , Takada T , Suzuki H , Onuki R , Hofmann AF , Sugiyama Y . Transport by vesicles of glycine‐ and taurine‐conjugated bile salts and taurolithocholate 3‐sulfate: a comparison of human BSEP with rat Bsep. Biochim Biophys Acta. 2005;54‐62. 10.1016/j.bbalip.2005.10.006 16332456

[28] Ijare OB , Bezabeh T , Albiin N , et al. Absence of glycochenodeoxycholic acid (GCDCA) in human bile is an indication of cholestasis: a ^1^H MRS study. NMR Biomed. 2009;471‐479. 19067402 10.1002/nbm.1355

[29] Maher J . LC‐MS/MS bile acid profiling as a biomarker for BSEP inhibition in Current trends in BSEP inhibition and perturbation to bile acid homeostasis as mechanisms of drug‐induced liver injury (Ryan Morgan GK‐U, Jonathan Maher, and John‐Michael Sauer ed), The Critical Path Institute's Predictive Safety Testing Consortium Series on current trends in BSEP inhibition and perturbation to bile acid homeostasis as mechanisms of drug‐induced liver injury, 2016 https://c-path.org/current-trends-in-bsep-inhibition-and-perturbation-to-bile-acid-homeostasis-as-mechanisms-of-drug-induced-liver-injury/.

[30] Linnet K , Mertz Nielsen A . Fasting and postprandial serum concentrations of glycine‐ and taurine‐conjugated bile acids in Crohn's disease. Scand J Gastroenterol. 1983;433‐438. 6673068 10.3109/00365528309181619

[31] Chen J , Deng W , Wang J , Shao Y , Ou M , Ding M . Primary bile acids as potential biomarkers for the clinical grading of intrahepatic cholestasis of pregnancy. Int J Gynaecol Obstet. 2013;5‐8. 10.1016/j.ijgo.2013.02.015 23562588

[32] Linnet K , Kelbaek H . The patterns of glycine and taurine conjugates of bile acids in serum in hepatobiliary disease. Scand J Gastroenterol. 1982;919‐924. 7156886 10.3109/00365528209181115

[33] Trottier J , Bialek A , Caron P , et al. Metabolomic profiling of 17 bile acids in serum from patients with primary biliary cirrhosis and primary sclerosing cholangitis: a pilot study. Dig Liver Dis. 2012;303‐310. 22169272 10.1016/j.dld.2011.10.025

[34] Woolbright BL , Dorko K , Antoine DJ , et al. Bile acid‐induced necrosis in primary human hepatocytes and in patients with obstructive cholestasis. Toxicol Appl Pharmacol. 2015;168‐177. 10.1016/j.taap.2015.01.015 PMC4361327 25636263

[35] Schulz S , Schmitt S , Wimmer R , et al. Progressive stages of mitochondrial destruction caused by cell toxic bile salts. Biochim Biophys Acta. 2013;2121‐2133. 23685124 10.1016/j.bbamem.2013.05.007

[36] Spivey JR , Bronk SF , Gores GJ . Glycochenodeoxycholate‐induced lethal hepatocellular injury in rat hepatocytes. Role of ATP depletion and cytosolic free calcium. J Clin Invest. 1993;17‐24. 8325981 10.1172/JCI116546 PMC293519

[37] Noto H , Matsushita M , Koike M , et al. Effect of high concentrations of bile acids on cultured hepatocytes. Artif Organs. 1998;300‐307. 9555961 10.1046/j.1525-1594.1998.05071.x

[38] Cepa S , Potter D , Wong L , et al. Individual serum bile acid profiling in rats aids in human risk assessment of drug‐induced liver injury due to BSEP inhibition. Toxicol Appl Pharmacol. 2017;204‐213. 29146462 10.1016/j.taap.2017.11.007

[39] Li X , Zhong K , Guo Z , Zhong D , Chen X . Fasiglifam (TAK‐875) inhibits hepatobiliary transporters: a possible factor contributing to fasiglifam‐induced liver injury. Drug Metab Dispos. 2015;1751‐1759. 26276582 10.1124/dmd.115.064121

[40] Morgan RE , van Staden CJ , Chen Y , et al. A multifactorial approach to hepatobiliary transporter assessment enables improved therapeutic compound development. Toxicol Sci. 2013;216‐241. 23956101 10.1093/toxsci/kft176

[41] Kenna JG , Taskar KS , Battista C , et al. Can BSEP inhibition testing in drug discovery and development reduce liver injury risk? ‐ An international transporter consortium perspective. Clin Pharmacol Ther. 2018;916‐932. 30137645 10.1002/cpt.1222 PMC6220754

[42] Yang K , Woodhead J , Morgan R , Watkins P , Howell B , Siler S . Mechanistic modeling with DILIsym predicts dose‐dependent clinical hepatotoxicity of AMG 009 that involves bile acid (BA) transporter inhibition (abstract). J Pharmacokinet Pharmacodyn. 2015;S21‐S22.

[43] Longo DM , Woodhead JL , Walker P , et al. Quantitative Systems Toxicology Analysis of In Vitro Mechanistic Assays Reveals Importance of Bile Acid Accumulation and Mitochondrial Dysfunction in TAK‐875‐induced Liver Injury. Toxicol Sci 2018 kfy253, 10.1093/toxsci/kfy253. [Epub ahead of print] PMC6358270 30289550

[44] Morgan RE . The AMG 009 Story: An opportunity to improve translational safety assessment in Current trends in BSEP inhibition and perturbation to bile acid homeostasis as mechanisms of drug‐induced liver injury (Ryan Morgan GK‐U, Jonathan Maher, and John‐Michael Sauer ed), The Critical Path Institute's Predictive Safety Testing Consortium Series on current trends in BSEP inhibition and perturbation to bile acid homeostasis as mechanisms of drug‐induced liver injury, 2016 https://c-path.org/current-trends-in-bsep-inhibition-and-perturbation-to-bile-acid-homeostasis-as-mechanisms-of-drug-induced-liver-injury/.

[45] Wolenski FS , Zhu AZX , Johnson M , et al. Fasiglifam (TAK‐875) alters bile acid homeostasis in rats and dogs: a potential cause of drug induced liver injury. Toxicol Sci. 2017;50‐61. 10.1093/toxsci/kfx018 PMC5414857 28108665

[46] Fattinger K , Funk C , Pantze M , et al. The endothelin antagonist bosentan inhibits the canalicular bile salt export pump: a potential mechanism for hepatic adverse reactions. Clin Pharmacol Ther. 2001;223‐231. 11309550 10.1067/mcp.2001.114667

[47] Markova SM , De Marco T , Bendjilali N , et al. Association of CYP2C9*2 with bosentan‐induced liver injury. Clin Pharmacol Ther. 2013;678‐686. 23863877 10.1038/clpt.2013.143 PMC3834031

[48] Lang C , Meier Y , Stieger B , et al. Mutations and polymorphisms in the bile salt export pump and the multidrug resistance protein 3 associated with drug‐induced liver injury. Pharmacogenet Genomics. 2007;47‐60. 17264802 10.1097/01.fpc.0000230418.28091.76

[49] Kaku K , Enya K , Nakaya R , Ohira T , Matsuno R . Long‐term safety and efficacy of fasiglifam (TAK‐875), a G‐protein‐coupled receptor 40 agonist, as monotherapy and combination therapy in Japanese patients with type 2 diabetes: a 52‐week open‐label phase III study. Diabetes Obes Metab. 2016;925‐929. 27178047 10.1111/dom.12693

[50] Otieno MA , Snoeys J , Lam W , et al. Fasiglifam (TAK‐875): mechanistic investigation and retrospective identification of hazards for drug induced liver injury. Toxicol Sci. 2018;374‐384. 28206647 10.1093/toxsci/kfx040

[51] Schadt HS , Wolf A , Pognan F , Chibout SD , Merz M , Kullak‐Ublick GA . Bile acids in drug induced liver injury: key players and surrogate markers. Clin Res Hepatol Gastroenterol. 2016;257‐266. 26874804 10.1016/j.clinre.2015.12.017

[52] Cook JC , Wu H , Aleo MD , Adkins K . Principles of precision medicine and its application in toxicology. J Toxicol Sci. 2018;565‐577. 30298845 10.2131/jts.43.565

