# Supplementary material for: Phase I study of PF‐04895162, a Kv7 channel opener, reveals unexpected hepatotoxicity in healthy subjects, but not rats or monkeys: clinical evidence of disrupted bile acid homeostasis
Source: Pharmacol Res Perspect. 2019 Feb 11;7(1):e00467. doi: 10.1002/prp2.467 (PMC6370995; doi:10.1002/prp2.467)
Supplement: Supplementary file 1 [file PRP2-7-e00467-s001.docx]

**Nonclinical Safety Assessment Summaries for PF‑04895162 (ICA‑105665):**

A summary assessment as it relates to liver function and changes are presented below for brevity. A synopsis of each nonclinical study that was used to support the IND filing and longer-term studies can be found in subsequent pages. All procedures performed on these animals were in accordance with regulations and established guidelines and were reviewed and approved by Pfizer Institutional Animal Care and Use Committee. All animals received human care according to or exceeding the criteria outlined in the “Guide for the Care and Use of Laboratory Animals” prepared by the National Academy of Sciences and published by the National Institutes of Health (NIH publication 86-23 revised 1985).

*Summary Safety Assessment Studies in Rats*

In an initial exploratory evaluation, Sprague-Dawley rats, administered PF‑04895162 once daily for 7 days at doses up to 300 mg/kg/day, experienced dose-dependent increases in ALT. However, there were no microscopic changes in the liver of high-dose animals associated with these changes. In a repeat study, Sprague-Dawley rats were administered PF‑04895162 twice daily for 7 days at doses up to 400 mg/kg/day but did not reproduce ALT findings noted in the earlier 7 day study. Only increased liver weight in female rats was noted. Total Cmax values in this study (presented as means) on Day 7 for male and female rats treated at 400 mg/kg/day were 7920 and 10260 ng/mL, respectively.

In the longer-term studies Sprague-Dawley rats were administered PF‑04895162 once daily for 28 days or 6 months at doses up to 100 mg/kg/day. No transaminase elevations were noted at any dose in the 28 day and 6 month study. Microscopic changes in liver were limited to centrilobular hypertrophy in females treated at 30 mg/kg/day and males and females treated at 100 mg/kg/day for 6 months. No treatment-related microscopic findings occurred in the liver of animals treated at 100 mg/kg/day for 28 days. Increased liver weights were noted in both studies. The drug was well absorbed into the systemic circulation of male and female rats following oral administration for 6 months. Total Cmax values on Day 1 in male and female rats treated at 100 mg/kg/day were 2463 and 5353 ng/mL, respectively. There was no evidence of accumulation of parent drug or any apparent induction of drug metabolizing enzymes by the end of the 6 month study.

*Summary Safety Assessment Studies in Monkeys*

Male and female cynomolgus monkeys were administered PF‑04895162 twice daily for 7 days at approximately 12 hours apart, with the dose split equally between the 2 daily doses. The highest administered does was 200 mg/kg/day. There were no clinical chemistry changes suggestive of liver effects. Increased liver weight was observed in both males and females in the 150 and 200 mg/kg/day groups. On Day 7 total Cmax values were >27000 ng/mL and >24000 ng/mL for males and females treated at 150 and 200 mg/kg/day, respectively.

A 28-day nasogastric toxicity study was conducted in cynomolgus monkeys at up to 100 mg/kg/day and a 9-month oral toxicity study with a 3-month interim sacrifice was conducted in cynomolgus monkeys at up to 100 mg/kg/day. No effect of treatment was seen in hematology, coagulation, clinical chemistry, macroscopic and microscopic pathology examinations. Mean absolute and relative increases in liver weights were seen in males at 30 and 100 mg/kg/day and in females at 100 mg/kg/day after 9 months of treatment with no clear microscopic correlate for this effect. An effect on lower mean values for parent AUC24 at the end of the 28-day study at 100 mg/kg/day, suggested the possibility for mild autoinduction of metabolism. After 9 months of treatment total Cmax values were approximately 29000 ng/mL for males and females treated at 100 mg/kg/day.

In summary, the compound advanced into clinical studies without any reproducible indication of transaminase elevations (observed in one short term rat study) and only nonadverse findings of centrilobular hepatocyte hypertrophy and increased liver weight in rat and monkey studies >1 month in duration where total Cmax concentrations were 6.9-15.1 and 81.6 µM in the rat (male-female) and monkey respectively. As is standard practice, total and fractionated bile acid determinations were not incorporated in the conduct of these studies.

| Study Title | | Species | Strain | | Group Size/Sex | | Dose Levels | | Route | | | Duration | | | GLP | | | | |  |
| --- | --- | --- | --- | --- | --- | --- | --- | --- | --- | --- | --- | --- | --- | --- | --- | --- | --- | --- | --- | --- |
| A Preliminary 7-Day Oral Toxicity Study in Rats with ICA‑105665 | | Rat | Sprague-Dawley | | 20 M  (vehicle) 10 M (ICA‑105665 dose groups) | | 0, 10, 30, 100, and 300 mg/kg | | PO | | | 7 days | | | NO | | |  |  |  |
| Observations: Mortality, general signs, body weight, gross pathology (including organ weights), serum chemistry, hematology and pathology (ICA‑105665 treated livers and kidneys only).  Results: Early in the study, clinical signs of tremor, splayed stance, altered gait, piloerection, and mild tremors were observed primarily at 300 mg/kg. Body temperature was decreased at 30, 100, and 300 mg/kg on Day 1 and at 300 mg/kg on Day 7. Body weight gain was suppressed on Days 3, 5, and 7 at 30, 100, and 300 mg/kg. Serum chemistry and hematology data showed several changes, most of which were believed not to be treatment-related but rather related to stress associated with decreased weight gain. ALT was increased in a dose-dependent manner but there was no correlation with liver tissue pathology. The only gross pathology finding was impacted feces in 1 animal at 100 mg/kg and 6 animals at 300 mg/kg. There were no microscopic changes in the liver or kidneys of high-dose animals. | | | | | | | | | | | | | | | | | | | | |
| ICA‑105665: A 7-Day Oral Toxicity Study in Rats | | Rat | Sprague-Dawley | 5 M, 5F Main Study  18M, 18F Toxicokinetic (TK) Study | | | | 0, 100, 200, and 400 mg/kg/day  (twice daily dosing) | | PO | | | 7 days | | YES | | | | |  |
| Observations: Mortality, general signs, body weight, food consumption, serum chemistry, hematology, toxicokinetics (Day 7) organ weights, gross and microscopic pathology.  Results: All main study animals survived to study termination. Clinical findings included marked hypothermia, decreased activity, ataxia, clonic convulsions, hypersensitivity to touch, lateral or ventral recumbency, prostration, tremors, high or low carriage, limb function impaired or lost, limbs splayed, black, red, and/or brown material around the eyes, black or brown material around the nose, red or yellow hair discoloration, skin cold to the touch, unkempt appearance, and slow or difficult breathing. These findings, along with the body temperature data were indicative of a test article-induced hypothermic condition. Decreases in body weight and food consumption were observed in treated males and females. There were no adverse test article-related effects on hematology parameters and no test article-related effects on coagulation or urinalysis parameters. Total protein, albumin, and globulins were dose-dependently decreased in males (10%) and females (17, 16, and 18%, respectively) most notably at 400 mg/kg/day. Values for globulins were affected to a lesser extent than total protein and albumin, and remained within expected normal ranges. Cholesterol was increased 1.72- to 2.56-fold in both sexes in all treated groups. Terminal body weights were decreased in males and females at 200 and/or 400 mg/kg/day. Organ weight effects were observed in males and/or females at 100, 200, and/or 400 mg/kg/day, including increased adrenal weights in males and females, increased liver weights in females, decreased spleen and thymus weights in males and females. Changes in weights in multiple organs, including the brain, kidneys, lung with bronchi, pituitary gland, prostate gland, mandibular and sublingual salivary glands, seminal vesicles and testes were considered to be associated with low body weights and decreased food consumption. However, direct test article-related effects on these organs cannot be excluded. In addition, there were decreased organ weight changes in the uterus and cervix of females at all dose levels. Microscopic examination was limited to the brain and no abnormalities were observed. A NOAEL could not be established for 7 days of twice daily dosing at 50, 100, and 200 mg/kg/dose (100, 200, and 400 mg/kg/day). | | | | | | | | | | | | | | | | | | | | |
| 28‑day Oral Gavage Toxicity and Toxicokinetic Study with ICA‑105665 in Rats with a 14-Day Recovery Period | Rat | | Sprague-Dawley | | | Main: 15 M; 15 F 10 M; 10 F 10 M; 10 F 15 M; 15 F   TK: 3M, 3F 9 M; 9 F 9 M; 9 F 9 M; 9 F | | 0 3 30 100   0 3 30 100 | | | PO      PO | | | 28 days,  14 day recovery for 5/sex in  0 and 100 mg/kg groups    28 days | | YES |  |  |  |  |
| Observations: Mortality; general signs; body weight; food consumption; body temperature, ophthalmology; clinical pathology (hematology, serum chemistry, coagulation and urinalysis on Day 29 and recovery); plasma drug levels (Days 1 and 28 in TK study animals); gross (including organ weights) and microscopic pathology. | | | | | | | | | | | | | | | | | | | | |
| Results: There was no mortality. Treatment-related clinical signs at 100 mg/kg included clear oral discharge (2/15 males), head tremors (2/15 females), rough haircoat (4/15 females), and few feces (6/15 females). There was no apparent treatment-related effect on body temperature. Mean body weight gain was decreased at 30 and 100 mg/kg; Day 28 mean body weights in males at 3, 30, and 100 mg/kg were 100, 92 and 90% of the mean control weight, respectively, and in females were 101, 97, and 95% of control. Mean food consumption was decreased at 30 and 100 mg/kg; Weeks 1-4 food consumption values in males at 3, 30, and 100 mg/kg were 99, 95 and 87% of the mean control food consumption, respectively, and in females were 101, 97, and 88% of control. There were no apparent treatment-related effects in the clinical pathology data. There were no obvious treatment-related gross pathology findings or mean organ weight changes. Mean liver weight in females at 3, 30, and 100 mg/kg were 111, 106, and 114% of control, respectively, but there were no treatment-related microscopic findings at 100 mg/kg/day. Since there was no histomorphologic correlate at 100 mg/kg for the organ weight findings, these organ weight differences were not considered to be related to treatment. The NOAEL for ICA‑105665 was 30 mg/kg/day. | | | | | | | | | | | | | | | | | | | | |
| ICA‑105665: 6-Month Oral Toxicity Study In Sprague Dawley (CD) Rats With A 4-Week Recovery Period | | Rat | Sprague Dawley | | 30 M, 30 F 20 M, 20 F 20 M, 20 F 30 M, 30 F  12 M, 12 F 12 M, 12 F 12 M, 12 F | | 0, 10, 30 and 100 (Main Study)    10, 30 and 100 (TK Animals) | | Oral (gavage) | | | 6-months | | | YES | | |  |  |  |
| Observations: Mortality, general signs, body weight, food consumption, clinical pathology, toxicokinetics (Day 1 and Months 3 and 6), organ weights, gross and microscopic pathology | | | | | | | | | | | | | | | | | | | | |
| Results: There were no test-article related deaths. Mean body weights were lower compared to controls in males at 30 and 100 mg/kg/day and in females at 100 mg/kg/day. Mean food consumption was less compared to controls during the entire 6-month study period in males and females at 100 mg/kg/day. In females only, a mild decrease in body temperature was seen on Days 1 and 91 at 30 and 100 mg/kg/day. Some clinical chemistry changes were seen including higher phosphorus, potassium, bilirubin, and cholesterol values and lower chloride and glucose levels in males and/or females at 30 and/or 100 mg/kg/day. Urinary volume and pH were higher compared to controls and urinary specific gravity was lower compared to control in males and females at most dose levels. Most all of these clinical chemistry and urinalysis changes had resolved during a 1‑month recovery period. No effect of treatment was seen in clinical findings, ophthalmoscopic, hematology, coagulation, and macroscopic pathology examinations. Organ weight changes included increased liver weights in both sexes at 30 and 100 mg/kg/day and decreased thymus gland weights in both sexes at 100 mg/kg/day. Microscopic changes were limited to centrilobular hepatocyte hypertrophy in the liver in females at 30 mg/kg/day and males and females at 100 mg/kg/day, thymus gland generalized lymphoid depletion in males and females at 100 mg/kg/day, and an increase in both the incidence and severity of ovarian atrophy at 30 and 100 mg/kg/day in animals at the terminal necropsy. All of these changes were essentially reversed by the 1-month recovery period. The NOAEL was therefore considered to be 30 mg/kg/day in males and 10 mg/kg/day in females. | | | | | | | | | | | | | | | | | | | | |
| Escalating Dose Range-Finding (DRF) and Maximum Tolerated Dose (MTD) Study with ICA‑105665 in Cynomolgus Monkeys | | Monkey | Cynomolgus | | Phase 1: 2M/2F   Phase 2: 2M/2F per group | | 0, 25, 50, 100 and 200   0, 100/200  (Days 1-3 and 5 at 100, Day 4 at 200) | | Nasogastric (NG)     NG | | | Single dose on Days 1, 4, 8, 11 and 15   5 days of dosing | | | YES | | |  |  |  |
| Observations: Mortality; general signs; body weight; food consumption; clinical pathology (hematology, serum chemistry, and urinalysis), plasma drug concentrations, body temperature, organ weights, gross pathology, and histopathology of Phase 1 liver sections only. | | | | | | | | | | | | | | | | | | | | |
| Results: Dosing Phase 1: Post-dosing vomitus was observed most prevalently following dosing at 100 and 200 mg/kg. Changes in the serum chemistry data noted after dosing at 100 or 200 mg/kg included slightly higher urea nitrogen, calcium, and/or electrolyte values that were likely related to the observed vomitus (e.g., mild dehydration). There were no other apparent effects of treatment, including gross findings at necropsy, organ weights, or histopathology findings in the liver sections.  Dosing Phase 2: Monkeys dosed once daily for 3 days at 100 mg/kg/day did not exhibit any apparent signs of toxicity, other than occasional post-dose vomitus in 1 male and female; therefore on Day 4, the dose was increased to 200 mg/kg/day. The morning following dosing at 200 mg/kg/day, all 4 treated monkeys were noted with clinical signs that included hyperactivity, ataxia, tremors, and dilated pupils, thus indicating that 200 mg/kg/day in a repeated-dose paradigm exceeded the MTD. Although variable, the 100 mg/kg/day monkeys tended to show greater reductions in body temperature relative to their pre-dose temperature compared to control monkeys at post-dose. | | | | | | | | | | | | | | | | | | | | |
| ICA‑105665: A 7-Day Oral Toxicity Study in Monkeys | | Monkey | Cynomolgus | | 3 M, 3F | | 0, 150 and 200 mg/kg/day (twice daily dosing) | | PO | | | 7 days | | | YES | | |  |  |  |
| Observations: Mortality, general signs, body weight, food consumption, serum chemistry, hematology, toxicokinetics (Days 1 and 7), organ weights, gross and microscopic pathology. | | | | | | | | | | | | | | | | | | | | |
| Results: All animals survived to the scheduled necropsy. However, due to marked clinical signs, 2 females and 1 male at 200 mg/kg/day were placed on drug holidays at various times during the 7-days of dosing. Treatment-related clinical findings observed at 150 and/or 200 mg/kg/day included decreased activity, ataxia, inappetence, stereotypy (hyper-reactivity, rigidity, jumping), tremors, loss of limb function, rapid eye movement, and eyelid partially/completely closed. There were no effects on body weight, body temperature, hematology, coagulation, clinical chemistry, or urinalysis parameters, or on macroscopic observations. Weight changes were seen in several organs, including increased liver weight in both males and females in the 150 and 200 mg/kg/day groups, and in males, dose-dependent increases in brain weight were seen at 150 and 200 mg/kg/day. Decreased thymus gland weight was seen in 150 and 200 mg/kg/day females. Microscopic examination was limited to the brain, and no abnormalities were observed. Based on the clinical findings observed in this study, a NOAEL could not be established for twice daily dosing for 7-days at 75 and 100 mg/kg/dose (150 and 200 mg/kg/day). | | | | | | | | | | | | | | | | | | | | |
| 28‑day Nasogastric Intubation Toxicity Study with ICA‑105665 in Cynomolgus Monkeys with a 14-Day Recovery Period | | Monkey | Cynomolgus | | 5 M; 5 F 3 M; 3 F 3 M; 3 F 5 M; 5 F 5 M; 5 F | | 0, 3, 10, 30, 100 | | Nasogastric (NG) | | | 28 days | | | YES | | |  |  |  |
| Observations: Mortality; general signs; body weight; food consumption; clinical pathology (hematology, serum chemistry, coagulation and urinalysis), plasma drug concentrations (Days 1 and 28), body temperature, electrocardiology, ophthalmology, organ weights, gross pathology and histopathology. | | | | | | | | | | | | | | | | | | | | |
| Results: All animals survived to their scheduled termination. Treatment-related clinical signs were noted at the 100 mg/kg/day dose level in both males and females, and included hunched posture (3/5 males and 3/5 females), body tremors (4/5 males and 3/5 females), ataxia (2/5 males and 2/5 females), hypoactivity (2/5 males), dilated pupils (4/5 males and 4/5 females), squinting (1/5 females), and vomitus (1/5 males and 2/5 females). Although vomitus was also noted at 30 mg/kg/day (1/5 males and 1/5 females), there were no other indications of treatment-related toxicity at this dose. No treatment‑related clinical signs were noted during the recovery phase. There were no direct treatment-related ophthalmic findings. Although variable, treatment at 30 and 100 mg/kg/day resulted in larger decreases in mean body (rectal) temperatures at 1, 2, and/or 4 hours post-dose, relative to pre-dose temperatures, on the first day of treatment as compared to the decreases in mean body temperature noted in the control animals. On dosing Day 28, no clear treatment-related effect on mean body temperature was apparent. There were no treatment-related body weight changes or electrocardiographic, clinical pathology, or anatomic pathology findings. The NOAEL was 30 mg/kg/day. | | | | | | | | | | | | | | | | | | | | |
| ICA-105665: 9-Month Oral Toxicity Study in Cynomolgus Monkeys with a 3-Month Interim Sacrifice and a 4-Week Recovery Period - 3 Month Interim Sacrifice | | Monkey | Cynomolgus | | 9 M; 9 F 7 M; 7 F 7 M; 7 F 9 M; 9 F | | 0, 10, 30 and 100 | | Oral (gavage) | | | 9-month with  3-month  (interim sacrifice) | | | YES | | |  |  |  |
| Observations: Mortality, general signs, body weight, food consumption, clinical pathology, toxicokinetics (Day 1, Month 3, Month 6, and Month 9), organ weights, gross and microscopic pathology | | | | | | | | | | | | | | | | | | | | |
| Results: One female at 100 mg/kg/day was found dead on Day 45 of the study. No significant clinical findings were seen prior to the death of the animal. The only significant microscopic findings in this animal were mild acute and granulomatous inflammation with intralesional plant material in the lungs. While the inflammation may have contributed to the demise of this animal, the lesions did not appear to be severe enough to be the definitive cause of death. All remaining animals survived during the study. Gross tremors and distended abdomens were observed sporadically in a few males and females at 100 mg/kg/day throughout the study. Ataxia, usually very slight, and mild incoordination were seen in a few animals very infrequently at 100 mg/kg/day. These clinical findings were not observed during the 4-week recovery period. No effect of treatment was seen in body weights, body temperatures, hematology, coagulation, clinical chemistry, ophthalmoscopic, electrocardiographic, macroscopic and microscopic pathology examinations. Mean absolute and relative increases in liver weights were seen in males at 30 and 100 mg/kg/day and in females at 100 mg/kg/day. While this increase in liver weights was likely test article-related, there was no clear microscopic correlate for this effect. The NOAEL was considered to be 30 mg/kg/day based upon the death of a single female animal at 100 mg/kg/day. | | | | | | | | | | | | | | | | | | | | |
